# Identification of the NADP^+^ Structural Binding Site and Coenzyme Effect on the Fused G6PD::6PGL Protein from *Giardia lamblia*

**DOI:** 10.3390/biom10010046

**Published:** 2019-12-27

**Authors:** Laura Morales-Luna, Abigail González-Valdez, Yudibeth Sixto-López, José Correa-Basurto, Beatriz Hernández-Ochoa, Noemí Cárdenas-Rodríguez, Rosa Angélica Castillo-Rodríguez, Daniel Ortega-Cuellar, Roberto Arreguin-Espinosa, Verónica Pérez de la Cruz, Hugo Serrano-Posada, Sara Centeno-Leija, Luz María Rocha-Ramírez, Edgar Sierra-Palacios, Alba Mónica Montiel-González, Yadira Rufino-González, Jaime Marcial-Quino, Saúl Gómez-Manzo

**Affiliations:** 1 Laboratorio de Bioquímica Genética, Instituto Nacional de Pediatría, Secretaría de Salud, Ciudad de México 04530, Mexico; lauraeloisamorales@ciencias.unam.mx; 2 Departamento de Biología Molecular y Biotecnología, Instituto de Investigaciones Biomédicas, Universidad Nacional Autónoma de México, Ciudad de México 04510, Mexico; abigaila@biomedicas.unam.mx; 3 Laboratorio de Modelado Molecular, Bioinformática y Diseño de Fármacos, Sección de Estudios de Posgrado e Investigación, Escuela Superior de Medicina, Instituto Politécnico Nacional, Ciudad de México 06720, Mexico; suyayq@gmail.com (Y.S.-L.); corrjose@gmail.com (J.C.-B.); 4 Laboratorio de Inmunoquímica, Hospital Infantil de México Federico Gómez, Secretaría de Salud, Ciudad de México 06720, Mexico; beatrizhb_16@comunidad.unam.mx; 5 Laboratorio de Neurociencias, Instituto Nacional de Pediatría, Secretaría de Salud, Ciudad de México 04530, Mexico; noemicr2001@yahoo.com.mx; 6 Consejo Nacional de Ciencia y Tecnología (CONACYT), Instituto Nacional de Pediatría, Secretaría de Salud, Ciudad de México 04530, Mexico; racastilloro@conacyt.mx; 7 Laboratorio de Nutrición Experimental, Instituto Nacional de Pediatría, Secretaría de Salud 04530, Mexico; dortegadan@gmail.com; 8 Departamento de Química de Biomacromoléculas, Instituto de Química, Universidad Nacional Autónoma de México, Ciudad de México 04510, Mexico; arrespin@unam.mx; 9 Departamento de Neuroquímica, Instituto Nacional de Neurología y Neurocirugía Manuel Velasco Suárez, S.S.A., Ciudad de México 14269, Mexico; veped@yahoo.com.mx; 10 Consejo Nacional de Ciencia y Tecnología (CONACYT), Laboratorio de Agrobiotecnología, Tecnoparque CLQ, Universidad de Colima, Carretera los Limones-Loma de Juárez, Colima 28629, Mexico; hjserranopo@conacyt.mx (H.S.-P.); scenteno0@ucol.mx (S.C.-L.); 11 Departamento de Infectología, Hospital Infantil de México Federico Gómez, Dr. Márquez No. 162, Col Doctores, Delegación Cuauhtémoc 06720, Mexico; luzmrr7@yahoo.com.mx; 12 Colegio de Ciencias y Humanidades, Plantel Casa Libertad, Universidad Autónoma de la Ciudad de México, Ciudad de México 09620, Mexico; edgar.sierra@uacm.edu.mx; 13 Centro de Investigación en Genética y Ambiente, Universidad Autónoma de Tlaxcala, Aut. San Martín Texmelucan-Tlaxcala Km 10.5, San Felipe Ixtlacuixtla, 90120 Tlaxcala, Mexico; amonicamg@yahoo.com; 14 México de Ciencias y Humanidades, Plantel Casa Libertad, Universidad Autónoma de la Ciudad de México, Ciudad de México 09620, Mexico; 15 Laboratorio de Parasitología Experimental, Instituto Nacional de Pediatría, Secretaría de Salud, Ciudad de México 04530, Mexico; yadirg@gmail.com

**Keywords:** NADP^+^ structural binding site, stability, G6PD, docking, *Giardia lamblia*

## Abstract

*Giardia lambia* is a flagellated protozoan parasite that lives in the small intestine and is the causal agent of giardiasis. It has been reported that *G. lamblia* exhibits glucose-6-phosphate dehydrogenase (G6PD), the first enzyme in the pentose phosphate pathway (PPP). Our group work demonstrated that the *g6pd* and *6pgl* genes are present in the open frame that gives rise to the fused G6PD::6PGL protein; where the G6PD region is similar to the 3D structure of G6PD in *Homo sapiens*. The objective of the present work was to show the presence of the structural NADP^+^ binding site on the fused G6PD::6PGL protein and evaluate the effect of the NADP^+^ molecule on protein stability using biochemical and computational analysis. A protective effect was observed on the thermal inactivation, thermal stability, and trypsin digestions assays when the protein was incubated with NADP^+^. By molecular docking, we determined the possible structural-NADP^+^ binding site, which is located between the Rossmann fold of G6PD and 6PGL. Finally, molecular dynamic (MD) simulation was used to test the stability of this complex; it was determined that the presence of both NADP^+^ structural and cofactor increased the stability of the enzyme, which is in agreement with our experimental results.

## 1. Introduction

*Giardia lamblia* is a unicellular, binucleated, and flagellated protozoan parasite [1,2]. Throughout its biological cycle, it presents two stages: the trophozoite (vegetative form), whose habitat is the small intestine of its host and is responsible for clinical manifestations named giardiasis; and the cyst (resistant and invasive form), responsible for the transmission of the parasite [3].

Giardiasis is the most common parasitic enteric disease in humans [4]. It has up to a quarter of a million cases worldwide [5], and developing countries are the most affected; the disease is widely distributed in rural areas due to lack of basic sanitary measures such as sewage and sewage disposal, population overcrowding problems, and the prevalence of bad hygiene habits, which favor the dispersion of the parasite [6]. The clinical manifestations of the disease can range from asymptomatic conditions to acute diarrhea or even malabsorption syndrome [7]. Children and immunocompromised patients are more exposed to the clinical consequences of infection by *Giardia lamblia* due to the high colonization capacity and the evasion of the immune system [8].

Phylogenetic studies based on ribosomal RNA (rRNA) and vacuolar ATPase categorize *G. lamblia* as a basal eukaryote [9,10]. Furthermore, it has been described that the metabolism of this parasite shows similar characteristics with anaerobic organisms, such as prokaryotes and archaea, as they contain no mitochondria [1], and the pentose phosphate pathway (PPP) is the main source of potential reducing agent, as nicotinamide adenine dinucleotide phosphate (NADPH). In addition, the PPP also provides ribose 5-phosphate, as a precursor to nucleic acids, and several intermediate metabolites, such as fructose 6-phosphate and glyceraldehyde 3-phosphate [11].

The PPP has two phases: the oxidative and non-oxidative phase. In the oxidative phase, the first and limiting enzyme is G6PD, which is responsible for generating one molecule of NADPH for each molecule of oxidized glucose [12]. G6PD is an enzyme that is present in all living organisms, and the active form exists in dimer–tetramer equilibrium whose stability is dependent of NADP^+^ concentration. In fact, it has been shown that human G6PD has a binding site located in the interface region of the protein known as the ‘structural NADP^+^ binding site’ that is important for the enzymatic activity [13,14,15]. Thus various working groups have evaluated the effect of NADP^+^ on the structural NADP^+^ binding site and have observed that mutations in human G6PD that occur near this site decrease the stability and dimerization of the enzyme, and in erythrocytes cause severe phenotypes, such as hemolytic anemia and chronic nonspherocytic hemolytic anemia (CNSHA) [16,17]. Furthermore, it was observed that the active site is located in the N-terminal domain (amino acids 27–200) and that it contains a Rossmann type folding (β–α–β dinucleotide binding site) [13,14,15].

Recent studies accomplished by Stover et al. [18] and Morales-Luna et al. [19] observed that the glucose-6-phosphate dehydrogenase (*g6pd*) and 6-phosphogluconolactonase (*6pgl*) genes were present in one open frame *g6pd*::*6pgl* gene, and that at the protein level it was possible to determine the existence of two functional domains present in the same (G6PD and 6PGL) sequenced regions. Furthermore, they observed that the amino acid sequence corresponding to the G6PD region on the fused G6PD::6PGL from *G. lamblia* was similar with the 3D structure of *H. sapiens* G6PD (PDB entry 2BH9). On the other hand, the G6PD protein from *G*. *lamblia*, despite being fused with the 6PGL, maintains the same structural conformation as the G6PD of other organisms, since it is also a dimer made up of two symmetrically located subunits and each monomer binds to a molecule of catalytic NADP^+^ coenzyme [19], in the Rossmann type folding domain. However, the existence of the structural NADP^+^ binding site on the fused G6PD::6PGL protein from *G. lamblia* was not demonstrated.

Based on the above, in this work by biochemical analysis and a computational approach, we demonstrate the presence of the structural NADP^+^ binding site on the fused G6PD::6PGL protein from *G. lamblia* and evaluate the effect of the NADP^+^ on the stability of the protein. By molecular docking, we determine the possible structural-NADP^+^ binding site, which is located between the Rossmann fold of G6PD and 6PGL. Moreover, it was possible to observe that the binding of the ADP moiety of NADP^+^ is bound in a similar way to that in humans, although the composition is not similar; thus, the structural NADP^+^ binding site of *Giardia lamblia* is partially conserved between humans and other species.

## 2. Materials and Methods

### 2.1. Purification of the Recombinant G6PD::6PGL Protein

The G6PD::6PGL protein was expressed and purified as previously reported by Morales-Luna et al. [19]. The *E. coli* BL21(DE3)Δ*zwf*::kan^r^ cells containing the pET-3a/*g6pd*::*6pgl* vector were induced with 1 mM of isopropyl-β-d-thiogalactoside (IPTG) for 18 h at 25 °C in Luria Bertani (LB) culture medium. The cells were pelleted by centrifugation, suspended in lysis buffer, and disrupted by sonication. Then, by centrifugation (10,000× *g* for 15 min at 4 °C), the crude extract was obtained and used for protein purification employing a 2′,5′-ADP Sepharose 4B affinity column (GE Healthcare, Chicago, IL, USA) and a Sephacryl 200 (16/60) gel filtration column (GFC) (GE Healthcare, Chicago, IL, USA) [19]. Purified G6PD::6PGL protein was analyzed in 12% SDS–PAGE gel [20] and stained with colloidal Coomassie Brilliant Blue (R-250) (Sigma-Aldrich, San Luis, MO, USA). The protein concentration was quantified according to Lowry et al. [21] using bovine serum albumin (BSA) as the standard.

### 2.2. Effect of the NADP^+^ Molecule on Protein Stability

#### 2.2.1. Thermal Inactivation Assay

The effect of the NADP^+^ molecule on the protein stability of the G6PD::6PGL protein from *G. lamblia*, was evaluated by thermal analysis. The G6PD::6PGL protein was adjusted to 0.2 mg/mL in buffer T (0.1 M Tris-HCl pH 7.35 plus 0.01 M MgCl_2_) with three different concentrations of the NADP^+^ molecule (0, 10, 100, and 500 μM) and incubated for 20 min in a temperature gradient ranging from 37 to 60 °C (Biorad T100 thermal cycler) as previously noted [22,23,24]. Then, the residual activity was measured spectrophotometrically and the *T*_50_ value (temperature at which the enzyme loses 50% of its initial activity) was determined. The residual activity in each condition measured at 37 °C was fixed as 100%. All thermal inactivation tests were performed in triplicate.

#### 2.2.2. Thermal Stability Assay

The effect of the NADP^+^ molecule on the G6PD::6PGL protein was evaluated by a thermostability test. The protein was adjusted to 0.5 mg/mL with buffer P (0.05 M potassium phosphate buffer pH 7.4) and incubated with three different concentrations of the NADP^+^ molecule (10, 100, and 500 µM). Thermal denaturation of the G6PD::6PGL protein was monitored at 222 nm in temperature scans ranging from 40 to 80 °C, increasing at a rate of 1 °C/2.5 min. The assay was performed in a spectropolarimeter (Jasco J-8190^®^, Easton, MD, USA), and the data were fit to the Boltzmann sigmoidal equation using the Origin program. The temperature at which 50% of the protein is folded and 50% is unfolded is expressed as melting temperature (*T*_m_) and was calculated as previously reported [25,26]. The experiment was performed in duplicate.

#### 2.2.3. Structural Analysis by Circular Dichroism (CD)

To evaluate the effect of the NADP^+^ molecule on the secondary structure of the recombinant G6PD::6PGL protein from *G. lamblia*, we performed an analysis of circular dichroism (CD) in a spectropolarimeter (Jasco J-810^®^, Inc., MD, USA). The G6PD::6PGL protein was adjusted to 0.5 mg/mL with buffer P in the absence and presence of three concentrations of the NADP^+^ molecule (10, 100, and 500 µM). Spectra were recorded at 25 °C in the ultra violet circular dichroism (UV–CD) ranging from 200 to 260 nm with a scanning speed of 50 nm·min^−1^. Spectra of blanks were subtracted from those that contained the protein. The experiments were performed in triplicate and the curves were plotted using the Origin program.

#### 2.2.4. Trypsin Digestion

The effect of the NADP^+^ molecule on the stability of the G6PD::6PGL protein was evaluated by the susceptibility to trypsin digestion in the presence of three different NADP^+^ concentrations (10, 100, and 500 µM). The protein was adjusted to 0.2 mg/mL in buffer T and incubated with different trypsin concentrations ranging from 0 to 1 mg/mL (0 to 190 units of trypsin) at 37 °C for 2 h [27]. Then, the residual activity was measured spectrophotometrically and expressed as a percentage of the activity of the same enzyme without trypsin. Furthermore, we evaluated the residual activity at time-course inactivation (from 0 to 120 min) incubated with 0.4 mg/mL or 76 units of trypsin in presence of three NADP^+^ concentrations (10, 100, and 500 µM). The residual activity was measured each 10 min and expressed as a percentage of the activity of the same enzyme without trypsin. The experiments were performed in triplicate.

#### 2.2.5. Analysis of the Stability of G6PD::6PGL in the Presence of Guanidine Hydrochloride (Gdn-HCl)

Finally, another approach to observe the protective effect of the NADP^+^ molecule on the stability of the G6PD::6PGL protein, we incubated it in presence of chaotropic agents as Gdn-HCl. The protein was adjusted to 0.2 mg/mL in buffer T, and incubated with distinct concentrations of Gdn-HCl (from 0 to 1 M) and three NADP^+^ concentrations (10, 100, and 500 µM) at 37 °C for 2 h [27]. Then, the residual activity was measured spectrophotometrically. The residual activity without Gdn-HCl was fixed as 100%. The experiments were performed in triplicate.

### 2.3. Determination of the K_d_ Value of G6PD::6PGL from G. lamblia

The dissociation constant for binding of NADP^+^ at the structural site in the G6PD::6PGL protein from *G. lamblia* was determined by protein fluorescence when it was titrated with the NADP^+^ molecule. For dissociation constant analysis, structural NADP^+^ was removed from the purified enzyme by buffer exchanged with buffer P, and enzyme concentration was subsequently adjusted to 0.1 mg/mL and incubated with different NADP^+^ concentrations (from 0 to 1000 µM), as reported previously [28]. Then, the scan spectrum of intrinsic fluorescence from 310–500 nm was obtained in a Perkin-Elmer LS-55 fluorescence spectrometer (Perkin Elmer, Wellesley, MA, USA) using an excitation wavelength at 295 nm, with 5 and 15 nm slits for excitation and emission, respectively. The final spectrum was the average of five scans; later, each spectrum was subtracted from the spectra of the blank (without protein plus NADP^+^). The data were fitted to non-linear regression calculations with the equation for a single binding constant using the Origin program. The experiment was performed in triplicate.

### 2.4. Alignment of the G6PD::6PGL Protein from G. lamblia

A total of 28 amino acid sequences were obtained from the National Center for Biotechnology Information (NCBI), and Swiss-Prot Multiple sequence alignment (MAS) of G6PDs amino acid sequences was performed with the BioEdit application using ClustalW using the parameter ‘auto’ option to select the most appropriate alignment strategy according to the program.

### 2.5. Molecular Docking of the NADP^+^ Molecule in G6PD::6PGL from G. lamblia

Autodock 4.2 software [29] was used for docking calculations. First, a blind docking study was performed to explore the whole protein, and further focused docking was conducted toward the possible binding site of the structural NADP^+^ (Q330, E339, L478, and R494) according to comparative structural analyses with human G6PD (PDB code: 2BH9), which was determined by using Chimera V.1.13 rc. The 3D structure of NADP^+^ was taken from PDB: 2BH9 and prepared for docking studies using AutoDock Tools 1.5.6, [29]. NADP^+^ was consider as flexible ligand during the molecular docking, while the protein was kept as rigid. On the other hand, for NADP^+^ Gasteiger charges were assigned, considering that at physiological pH is charged (−3). The G6PD::6PGL model was obtained from a previous work [19] (Supplementary Materials g6pd.6pgl.pdb) and refined using the ModRefiner server [30] (Supplementary Materials g6pd_clean-rama.pdf). For G6PD::6PGL only polar hydrogens were considered, which are hydrogen atoms that are bonded to electronegative atoms (O, N, S) that are capable of forming hydrogen bonds; and charges were assigned Kollman united-atom library, which fits the electrostatic potential to points selected on a set of concentric spheres around each atom; the sum of all atomic charges is equal to that of the overall charge of the system [31] and the AD4.1_bound forcefield was employed in molecular docking [29].

Molecular docking was carried out with a grid box with a dimension of 126 Å^3^ for blind docking and 60 Å^3^ for focused docking and a grid spacing of 0.375 Å^3^. The Lamarckian genetic algorithm was used to score sampling with a randomized population of 100 individuals and energy evaluations of 1 × 10^7^; 100 runs were performed. The most populated cluster G6PD::6PGL–NADP^+^ complex with the lowest free energy values was taken as the starting conformation to carry out the molecular dynamics (MD) simulations, as described below. Docking results were analyzed using Autodock Tools 1.5.6 [29], and figures were processed with Pymol v.099 [32] (the PyMOL Molecular Graphics System, Version 2.0 Schrödinger, LLC, New York, NY, USA) and Chimera UCSF V1.13rc [33], and a two dimension (2D) map of the G6PD::6PGL–NADP^+^ complex interactions was retrieved from Discover Studio V17.2 (Dassault Systèmes BIOVIA, Discover Studio, V17.2, Dassault Systèmes: San Diego, CA, USA, 2019).

### 2.6. Molecular Dynamics (MD) Simulations of the NADP^+^ Molecule in G6PD::6PGL from G. lamblia

MD simulations were carried out with the AMBER 16 software package [34] using the ff14SB forcefield [35]. NADP^+^ parameters were obtained from a previous work [36]. Four systems were studied: the Apo form of G6PD::6PGL (APO) with the NADP^+^ cofactor (cofactor), with the structural NADP^+^ (structural), and with both NADP^+^ as cofactor and as structural NADP^+^ (holo). Each of the systems were solvated using the explicit TIP3P water model [37] and centered into a orthorhombic box of 12.0 Å. The system was neutralized by adding the corresponding Na^+^. Systems were minimized through 2500 steps of steepest descent and 2500 steps of conjugate gradients. Then, they were equilibrated through 500 picoseconds (ps) of heating and 500 ps of density equilibration with weak restraints on the complex, followed by 2 nanoseconds (ns) of constant pressure equilibration at 310 K. MD simulations was carried out through 20 ns for the systems, under periodic boundary conditions, and using an NPT ensemble at 310 K. The electrostatic term was described through the particle mesh Ewald method [38], using a 10.0 Å cut-off radio; that was chosen for Van der Waals interactions. The SHAKE algorithm was used to constrain bond lengths at their equilibrium values and the time step was set to 2.0 fs. Temperature and pressure were maintained with the weak coupling algorithm using coupling constants τT and τ*p* of 1.0 and 0.2 ps, respectively (310 K, 1 atm). Trajectories were analyzed using AmberTools 16. Structural analysis of the structures were performed using Chimera V1.13rc [33] and PyMOL v0.99 [32] (The PyMOL Molecular Graphics System, Version 2.0 Schrödinger, LLC).

## 3. Results and Discussion

### 3.1. Effect of the NADP^+^ Molecule on Protein Stability

The G6PD::6PGL protein was purified according to methods reported previously by Morales-Luna et al. [19]. Furthermore, a homology model of the fused G6PD::6PGL protein was obtained (Supplementary Materials file Gl-G6PD-6PGL_Minimized.pdb). However, the effect of the NADP^+^ molecule on the fused G6PD protein of *G. lambia* was not demonstrated.

#### 3.1.1. Thermal Inactivation Assay

**Figure 1:**
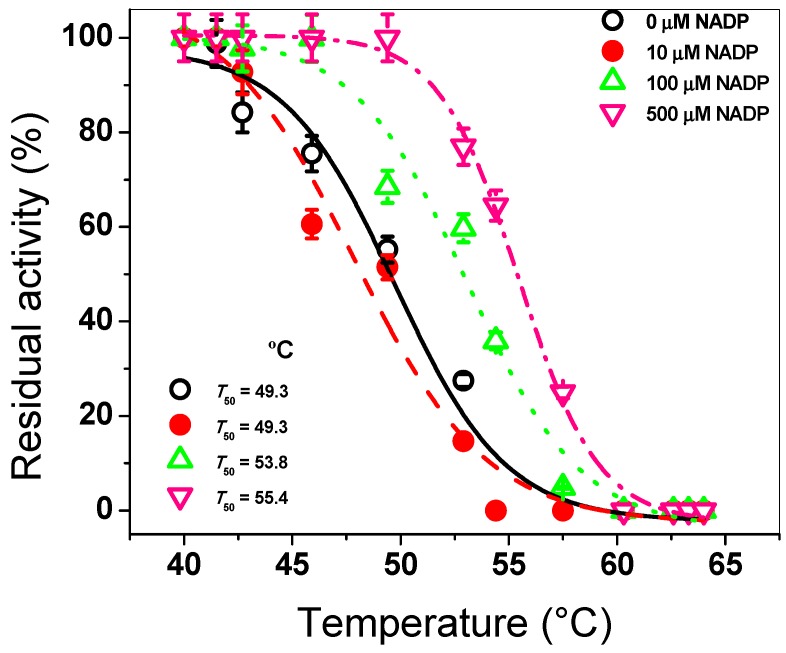
Thermal inactivation assays of recombinant G6PD::6PGL protein from *G. lamblia*. The protein was incubated in the absence or presence of NADP^+^. In all the cases, the protein was adjusted to 0.2 mg/mL, and 200 ng of total protein was used to measure the residual activity after being incubated in a temperature gradient ranging from 37 to 60 °C for 20 min. The residual activity measured at 37 °C was fixed as 100%. Experiments were performed in triplicate; standard errors were less than 5%.

To evaluate the effect of the NADP^+^ molecule on the G6PD::6PGL enzyme from *G. lamblia*, we performed a thermal inactivation assay, which has been widely used to evaluate the stability of the active site of the G6PDs proteins from diverse organisms [22,23,24,25,26,27]. The fused G6PD::6PGL protein was incubated with three NADP^+^ concentrations (10, 100, and 500 μM) in a temperature gradient from 37 to 60 °C. As seen in Figure 1, when the enzyme was incubated with 10 µM of NADP^+^, the protein showed a *T*_50_ of 49.3 °C, which was similar to that without NADP^+^. However, when the G6PD::6PGL protein was incubated with 100 and 500 µM of NADP^+^, the *T*_50_ determined were 53.8 °C and 55.4 °C, respectively. These results indicated that the G6PD::6PGL protein became more resistant to temperature with respect to the G6PD::6PGL protein without NADP^+^, showing a shift of 6 °C in the presence of NADP^+^ compared to the G6PD::6PGL protein in the absence of the molecule (Figure 1). This shift of 6 °C could indicate the presence of the structural NADP^+^ binding site in the G6PD::6PGL protein. This protective effect was observed in recombinant human G6PD when the recombinant human G6PD enzyme is incubated with different NADP^+^ concentrations (from 0 to 500 µM); the recombinant human G6PD enzyme was more resistant to temperature in the presence of this molecule, where a shift of 10 °C in the *T*_50_ compared to that without NADP^+^ was observed [22,23,24,25,26,27,28,39].

#### 3.1.2. Thermal Stability Assay

**Figure 2:**
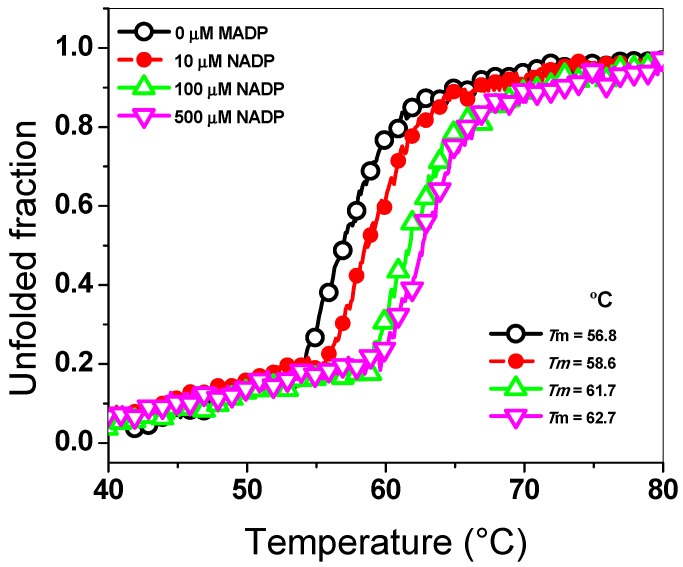
Thermal stability of the G6PD::6PGL protein from *G. lamblia*. Changes in the CD signal at 222 nm were monitored when the temperature was increased progressively from 40 to 80 °C at 1 °C/2.5 min. The protein was adjusted to 0.5 mg/mL in buffer P. The experiment is representative of duplicate experiments.

To evaluate the effect of the NADP^+^ molecule on the global stability and unfolding assay of the G6PD::6PGL enzyme, we determined the structural changes in the G6PD::6PGL protein using CD analysis at 222 nm when the temperature was increased. As seen in Figure 2, we observed a two-state process (native and unfolded protein) when the temperature was increased, and the temperature at which half of the secondary structure (α–helices) was unfolded was defined as *T*_m_. The *T*_m_ value determined in the absence of NADP^+^ was 56.8 °C, while those in the presence of 10, 100, and 500 µM of the NADP^+^ molecule were 58.6, 61.7, and 62.7 °C, respectively. These results indicated that the G6PD::6PGL protein became more resistant to unfolding by temperature compared to that incubated without the NADP^+^ molecule, where a shift of 6 °C in the *T*_m_ value was observed in the presence of NADP^+^ (500 µM) compared to that in the absence of the same molecule. This shift of 6 °C, could be due to the presence of the structural NADP^+^ binding site in the G6PD::6PGL of *G. lamblia*. Based on the thermal inactivation and thermal stability assays both in the absence and presence of the NADP^+^ molecule, we observed that the G6PD::6PGL protein in the presence of the NADP^+^ molecule became more resistant to temperature both in its active site (thermal inactivation) and in its thermal stability (global stability of the protein). These experimental results gave us a first approach in the elucidation of the existence of the structural NADP^+^ binding site in the G6PD::6PGL of *G. lamblia*, because it was possible to detect the protective effect of this molecule as previously observed in human G6PD enzyme [22,23,24,25,26,27,28,39].

#### 3.1.3. Structural Analysis by CD

**Figure 3:**
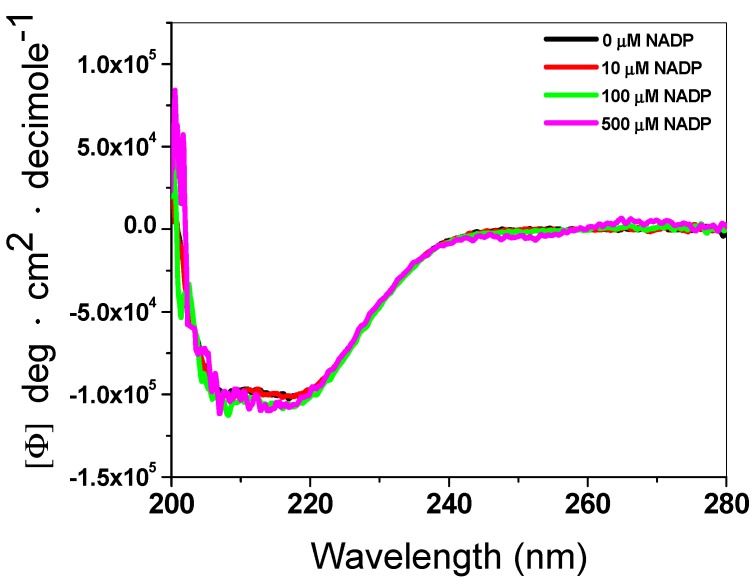
Far-UV circular dichroism (CD) spectra of the G6PD::6PGL protein. Far-UV CD spectra were performed in a spectropolarimeter (Jasco J-810^®^). The protein concentration was 0.5 mg/mL in buffer P. The spectra of blanks were subtracted from those that contained the protein. The experiment is representative of duplicate experiments.

To evaluate the effect of the NADP^+^ molecule in the secondary structure of the recombinant enzyme G6PD::6PGL, we performed an CD analysis in the absence or presence of three different concentrations of NADP^+^. As can be seen in Figure 3, the ultra violet circular dichroism (UV–CD) spectra did not show changes in the secondary structure of the protein (α–helices and β–sheets), either in the absence or presence of the NADP^+^ molecule. This result indicated that the NADP^+^ molecule did not alter the folding of the protein at the level of the secondary structure, and the protective effect observed on the thermal inactivation and thermal stability assays could be due to the presence of the structural NADP^+^ binding site in the G6PD::6PGL of *G. lamblia*.

#### 3.1.4. Trypsin Digestion

**Figure 4:**
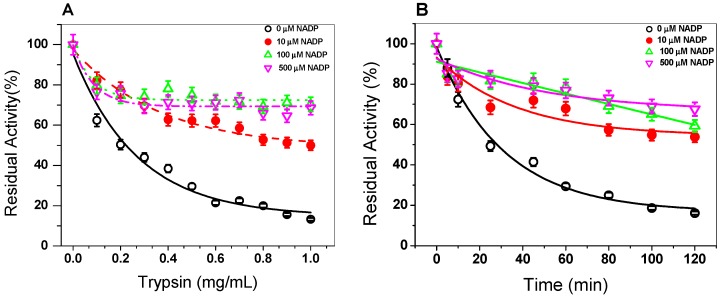
Trypsin digestion of G6PD::6PGL from *G. lamblia*. (**A**) The protein adjusted to 0.2 mg/mL was incubated with three NADP^+^ concentrations (10, 100, and 500 μM) and different concentrations of trypsin (0 to 1 mg/mL (0 to 190 units). The protein was incubated for 2 h at 37 °C and the residual activity was measured. (**B**) Time-course protease digestion of the G6PD::6PGL protein. The protein adjusted to 0.2 mg/mL was incubated with trypsin (0.4 mg/mL or 76 units) at 37 °C. At the times indicated in the ordinate axis, the reaction was arrested by the addition of PMSF 5 mM and the residual activity was measured under standard conditions. The experiment was representative of a triplicate; standard errors were less than 5%.

In order to evaluate the effect of the NADP^+^ molecule on the stability of the G6PD::6PGL protein, we evaluated the susceptibility to trypsin digestion in the absence or presence of three different concentrations of NADP^+^. As seen in Figure 4A, when the trypsin concentration was increased (from 0 to 1 mg/mL) (from 0 to 190 units), the native protein became more susceptible to trypsin digestion, where about 90% of the initial activity was lost when the enzyme was incubated with 1 mg/mL of trypsin (190 units). However, when the G6PD::6PGL protein was incubated with 10 µM of NADP^+^, the enzyme became more resistant, where 40% of the residual activity was recovered compared to the G6PD::6PGL protein without the NADP^+^ molecule with 1 mg/mL of trypsin (190 units). Furthermore, the G6PD::6PGL protein only lost 30% of the initial activity when it was incubated with 100 and 500 µM of NADP^+^ in the presence of 1 mg/mL of trypsin (190 units). These results again indicate that the G6PD::6PGL protein became more resistant to trypsin digestion in the presence of the NADP^+^ molecule. It is important to note that the trypsin digestions assay has been widely used to evaluate the stability of proteins, especially in the human G6PD and their variants, where it has been observed that in the presence of the NADP^+^ molecule, the protein has become more resistant to protease digestion compared to that without NADP^+^ [25,27].

In addition, the effect of the NADP^+^ molecule was evaluated by time-course inactivation of the G6PD::6PGL protein. As seen in Figure 4B, the G6PD::6PGL protein incubated with 0.4 mg/mL of trypsin (76 units) in the absence of the NADP^+^ molecule displayed a high susceptibility to trypsin digestion, losing 50% of its residual activity after 25 min of incubation; meanwhile, the G6PD::6PGL protein was incubated in the presence of NADP^+^ (10, 100, and 500 μM), and the enzymes became more resistant to protease degradation, where at 120 min, the enzyme lost 20% of its initial activity, which indicated that the addition of NADP^+^ showed a protective effect against trypsin digestion.

#### 3.1.5. Stability of G6PD::6PGL in the Presence of Gdn-HCl

**Figure 5:**
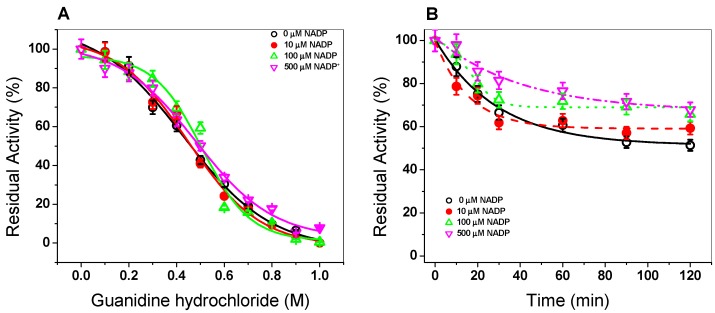
Stability of the G6PD::6PGL protein. (**A**) Activity of the G6PD::6PGL protein in the presence of Gdn-HCl and three NADP^+^ concentrations. The residual activity measured at 37 °C without Gdn-HCl was fixed as 100%. (**B**) Time-course residual activity of the G6PD::6PGL protein in the presence and absence of the NADP^+^ molecule and incubated with 0.45 M of Gdn-HCl. At the indicated times, aliquots were withdrawn and residual activity was measured. In both assays, the G6PD::6PGL protein was adjusted to 0.2 mg/mL in buffer P. Experiments were performed in triplicate; standard errors were less than 5%.

Finally, we evaluated the effect of the NADP^+^ molecule on the G6PD::6PGL protein in presence of chaotropic agents such as Gdn-HCl (ranging 0 to 1 M) and three different NADP^+^ concentrations (10, 100, and 500 µM). As seen in Figure 5A, a sigmoidal decay of the activity of the G6PD::6PGL protein in presence of Gdn-HCl (0–1 M) was observed as it was increased. At 0.1 M of Gdn-HCl, no effect was observed on the activity of G6PD::6PGL, whereas from 0.4 M to 0.5 M, the activity decreased around 50%, and no activity was detected at 1 M of Gdn-HCl. When the G6PD::6PGL protein was incubated with the NADP^+^ molecule, the protein showed the same effect as in the absence of NADP^+^. This result indicates that the enzyme was not protected by the NADP^+^ molecule. The Gdn-HCl_1/2_ value (Gdn-HCl concentration at which the enzymes lose 50% of original activity after 2 h at 37 °C) was determined to be 0.45 M of Gdn-HCl, both in the absence and presence of NADP^+^.

Given that no protective effect was observed when the enzyme was incubated with Gdn-HCl for 2 h, we decided to evaluate the activity in a time-course inactivation of the G6PD::6PGL protein (0 to 120 min) incubated with Gdn-HCl_1/2_ (0.45 M). As seen in Figure 5B, when the enzyme was incubated with high NADP^+^ concentrations (100 and 500 µM) the enzyme became more resistant to the chaotropic agent because the enzyme recovered 20% of the activity compared to that without NADP^+^ (Figure 5B). This slight recovery in the residual activity again indicated to us that the NADP^+^ molecule provoked the protein to be more resistant to the chaotropic agent and could indicate that the molecule was interacting with the protein and causing a protective effect.

Based on all the trials, we evaluated the effect of the NADP^+^ molecule on the stability of the protein and found that when the G6PD::6PGL protein was incubated with the NADP^+^ molecule, the enzyme was more resistant to temperature both in its active site (thermal inactivation) and in its thermal stability (global stability of the protein). Furthermore, we evaluated the effect of the NADP^+^ molecule in the presence of trypsin and Gdn-HCl, and again we observed that when the protein was incubated with 500 µM of NADP, it became more resistant to denaturation by protease or to chaotropic agents such as guanidine. These experimental results gave us a first approach in the elucidation of the existence of the structural NADP^+^ binding site in the G6PD::6PGL of *G. lamblia*, because it was possible to detect the protective effect of this molecule on the G6PD::6PGL protein.

### 3.2. Determination of the Ligand Dissociation Constant (K_d_) of Structural NADP^+^

**Figure 6:**
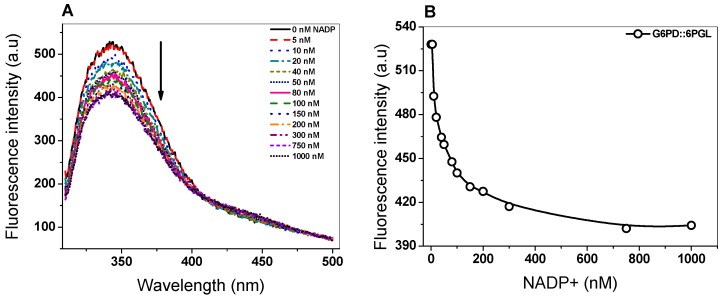
Determination of the *K*_d_ of structural NADP^+^. The G6PD::6PGL protein was adjusted to 0.1 mg/mL in buffer P and titrated with NADP^+^. (**A**) The fluorescence signal intensity was recorded in a Perkin-Elmer LS-55 fluorescence spectrometer after each addition using excitation wavelength at 295 nm, with 5 and 5 nm slits for excitation and emission, respectively. (**B**) Data of intrinsic fluorescence obtained by subtracting the blank from the values of total fluorescence intensity were fitted to non-lineal regression calculations. The experiments were performed in triplicate; standard errors were less than 5%.

Previously, within the recombinant human G6PD there was demonstrated the existence and the stoichiometric content of one molecule/subunit of structural NADP^+^, which occupied a second site different to catalytic NADP^+^ [28]. Moreover, Au and collaborators [15], in the crystal structure of human G6PD (Canton), demonstrated the presence of a structural NADP^+^ binding site located in the dimer interface of β–sheets of each subunit in the C terminus region. Furthermore, it was demonstrated that this molecule confers structural stability and is necessary for dimerization on the G6PD protein [40,41,42]. Due to thermal inactivation and thermal stability assays in the G6PD::6PGL protein, it was demonstrated that the NADP^+^ molecule gives a protective effect on the protein, and we determined the ligand dissociation constant (*K*_d_) value for the NADP^+^ at the structural site. The *K*_d_ was determined by protein fluorescence when it was incubated with NADP^+^. As seen in Figure 6A, the freshly G6PD::6PGL stripped enzyme gave a maximal fluorescence emission spectrum of 528 arbitrary units (a.u.) with a maximum peak at 344 nm (Figure 6B). As the concentration of the NADP^+^ molecule was increased, the fluorescence intensity was partially quenched until 404 arbitrary units (a.u.) in the presence of 300 µM of NADP^+^. Furthermore, we plotted the titration data of the maximal fluorescence emission versus NADP^+^ concentration and calculated a value of the dissociation constant of 63.2 nM (Figure 6B). This *K*_d_ value determined for the structural NADP^+^ differed in its affinity for the catalytic NADP^+^, where the *K*_d_ value was 219-fold lower in terms of its affinity for catalytic NADP^+^ (*K*_m_ = 13.9 µM) [19]. It is interesting to note that these results were in concordance with those reported by Wang et al. [28], where the *K*_d_ value for structural NADP^+^ in the recombinant human G6PD was 37 nM, which was 200-fold lower than 7.8 µM for catalytic NADP^+^ [43]. These results indicated that the G6PD::6PGL enzyme from *G. lamblia* had the ability to bind to the structural NADP^+^ and confirmed the results previously observed in thermal inactivation and thermal stability, where a protective effect in the protein was observed.

### 3.3. Alignment of the G6PD::6PGL Protein

Since the G6PD::6PGL enzyme from *G. lamblia* has the ability to bind one additional NADP^+^ (structural) and shows a protective effect from this molecule, we performed a multiple sequence alignment with eukaryotic G6PDs amino acid sequences (retrieved from Swiss-Prot and NCBI) to identify the amino acid residues responsible for the structural NADP^+^ binding. Previously, Kokata et al. [14] identified the amino acid residues involved in structural NADP^+^ binding on human G6PD. As seen in Supplementary Materials Figure S1, the analysis involved 28 amino acid sequences of eukaryote organisms, and it was possible to find all the amino acids involved in the binding of the structural NADP^+^. The amino acids that participated in the binding of structural NADP^+^ in the human G6PD were: Lys238, Lys366, Arg370, Arg393, Tyr401, Lys403, Asp421, Thr423, Arg487, Tyr503, and Trp509 (amino acid number corresponding to human G6PD). When the alignment was performed, these amino acids (indicated by asterisks) were conserved in different species; however, the amino acids from *G. lamblia* were different, as showed in the Supplementary Materials Figure S1.

Although the G6PD::6PGL from *G. lamblia* showed changes in the amino acids that participate in the binding of structural NADP^+^ in human G6PD; we observed through experimental trials, as shown in this work, that the G6PD::6PGL protein is stabilized by the NADP^+^ molecule, which suggests to us that the protein has the ability to bind the structural NADP^+^. These changes in the amino acids that interact in the binding of the structural NADP^+^ in the G6PD::6PGL of *G. lamblia* and human G6PD could be explained by the difference of ligand *K*_d_ of structural NADP^+^. Furthermore, this protective effect is in agreement with that previously reported by Gómez-Manzo et al. [22,23], where it was observed through in site-directed mutagenesis in amino acids involved in the union of structural NADP^+^, as in the Class I Nashville (R393H) and Seattle (D282H) variants, that although there were amino acid changes in these variants, it was possible to maintain the binding to NADP^+^, and that when these G6PD variants were incubated with the NADP^+^ molecule, the enzyme was stabilized by this molecule [22,27]).

### 3.4. Molecular Docking of the NADP^+^ Molecule in G6PD::6PGL from G. lamblia

**Figure 7:**
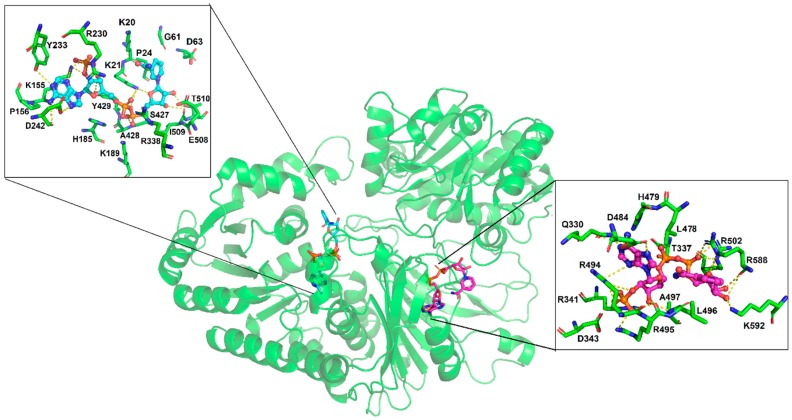
Molecular docking of NADP and the G6PD::6PGL model; the NADP^+^ cofactor binding pose is shown as cyan sticks and the NADP^+^ structural binding pose is depicted as magenta sticks. The interacting residues are depicted in a box, in the left panel the NADP^+^ cofactor and in the right panel structural NADP^+^.

Molecular docking was performed in order to explore the binding site of NADP^+^ on G6PD::6PGL (cofactor and structural). Under the blind docking procedure, we were able to predict the cofactor NADP^+^ binding site, where NADP^+^ made non-bonding interactions (hydrogen bonds) with residues belonging to the catalytic sites Y233, D242, A428, Y429, and I509, and made a salt bridge with K21, K155, H185, R230, and R338 and hydrophobic interactions with K20, P24, G61, D63, P156, K189, S427, E508, and T510, with a favorable free energy of binding of −9.12 kcal/mol. As a second exploration, it was possible to predicted the possible binding site for the structural NADP^+^, it showed lesser free energy of binding compared to cofactor NADP^+^ (−8.13 kcal/mol) (Supplementary Materials docking_both.pdb). It was found that structural NADP^+^ was bound to a region closer to the corresponding human one; however, in *G. lamblia*, the 6PGL domain was near to this site, causing a steric impediment. Therefore, only the ADP portion was bound with similar binding pose to that in humans, while the ribose and nicotinamide portion were bound to residues of G6PD and 6PGL, protruding to the exterior (Figure 7). It formed hydrogen bonds with Q330, T337, D484, A497, and K592; salt bridges with R341, R494, R495, R502, and R588; and hydrophobic interactions with D343, L496, L478, and H479. Through a structural alignment, it was possible to identify that the interacting residues were spatially placed in the same position as that of the corresponding human structural NADP^+^ binding site (PDB entry: 2BH9) [14], such as HuG6PD R357 → G6PD::6PGL Q330, HuG6PD R370 → G6PD::6PGL D343, HuG6PD R487 → G6PD::6PGL L478, and HuG6PD Y503 → G6PD::6PGL R494, which are residues that housed the nicotinamide and sugar portions (ADP moiety); these differences showed that these chemical moieties adopted different spatial conformation (Figure 7). In summary, structural NADP^+^ was able to be bound to the so-called structural site, but with different binding poses and different residues than those described in humans. Finally, it is important to highlight the importance of the presence of the structural domain in the G6PD enzyme throughout evolution, since despite the phylogenetic position of eukaryotes, this “structural NADP^+^ binding site” is present in eukaryotes of early divergence, such as *G. lamblia*; thus, we could elucidate that the presence of the structural NADP^+^ binding site was implemented as a mechanism that allows the G6PD enzyme to remain stable for a longer period of time, even under stressful conditions in the environment.

### 3.5. MD Simulations of the NADP^+^ Molecule in G6PD::6PGL from G. lamblia

**Figure 8:**
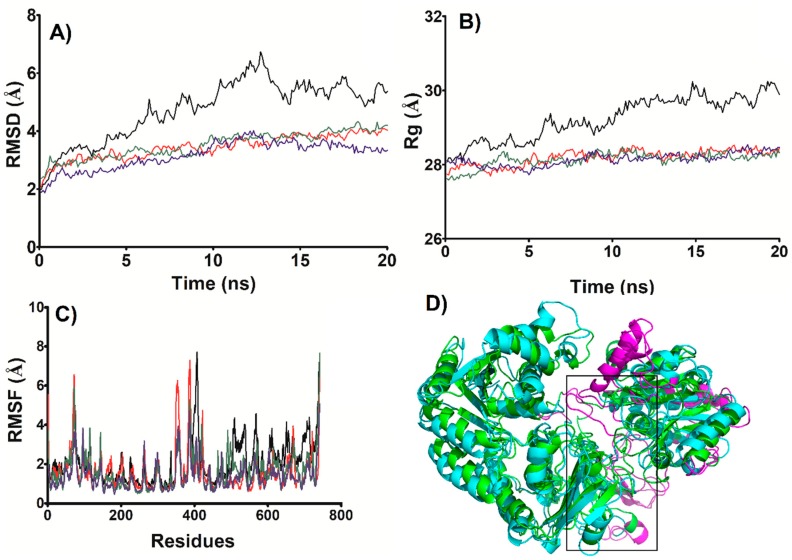
Structural analysis of the molecular dynamic (MD) simulation of the G6PD::6PGL systems. (**A**) Root mean square deviation (RMSD), (**B**) radius of gyration (Rg), (**C**) root mean square fluctuation (RMSF), and (**D**) superimposition of the G6PD::6PGL model and the most populated cluster conformation of APO G6PD::6PGL retrieved from MD simulation. In the graph, the Apo system is depicted as a black line, the structural system is depicted as a red line, the cofactor system is depicted as a green line, and holo is depicted as a blue line. Native conformation of apo G6PD::6PGL is depicted as a green cartoon and the most populated cluster conformation of apo system is depicted as a cyan ribbon; regions with higher fluctuations in the apo system are depicted as magenta ribbons.

In order to explore the stability of the G6PD::6PGL (structure) complex found by docking, MD simulation was performed, exploring four systems: the Apo-G6PD::6PGL (Apo) (Supplementary Materials cluster.apo.pdb), cofactor NADP^+^–G6PD::6PGL (cofactor) (Supplementary Materials cluster.cofactor.pdb), structural NADP^+^–G6PD::6PGL (structural) (Supplementary Materials cluster.structural.pdb), and a fourth system with both structural and cofactor NADP^+^–G6PD::6PGL (Holo) (Supplementary Materials cluster.holo_both.pdb). According to root mean square deviation (RMSD), the system reached stability after 10 ns (Figure 8A) and the same behavior was also observed with Rg (Figure 8B). The Apo showed an RMSD of 5.56 ± 0.38 Å, while the Cofactor (3.90 ± 0.16 Å), Structural (3.77 ± 0.19 Å), and Holo (3.56 ± 0.18 Å) systems depicted values less than 4.0 Å. A similar tendency was observed with Rg, where Apo depicted the higher Rg value (29.75 ± 0.22 Å), and the other systems did not show significant Rg differences and converged around 28 Å (structural: 28.33 ± 0.1 Å; cofactor: 28.23 ± 0.09 Å; and Holo: 28.24 ± 0.12 Å). Both RMSD and Rg indicated that the Apo system was less stable than the systems that possessed NADP^+^ as part of their structure. As for the degree of compaction (Rg), the absence of NADP^+^ implied that the Apo enzyme was more expanded in comparison with that possessing NADP^+^.

In terms of fluctuations (Figure 8C), the Apo form depicted fluctuations higher than 3.0 Å, mainly in regions belonging to the σ-helices nearer where the structural NADP^+^ was allocated, as well as loops located at G6PD and 6PGL interfaces (351–358, 383–413, 419–423, and 505–511); however, most of the regions with higher fluctuations were in the 6PGL domain (527–540, 607–614, 649–653, 694–715, and 563–571) (Figure 8D).

Interestingly, when cofactor NADP^+^ was added, the system became stabilized and overall fluctuations decreased, especially in 6PGL domain. In the structural system, when only structural NADP^+^ was present, the stabilization of 6PGL was more evident than that produced by cofactor NADP^+^, since fluctuations were smaller. Fluctuations in G6PD were almost equal to those observed in the APO system, except in two loops that joined two alpha helices localized into the Rossmann fold (351–369 and 386–390), whose residues did not interact with the structural NADP^+^ even though they did interact with HuG6PD. However, in the Holo system, when both NADP^+^ were present, the stabilization of the whole system was evident, since fluctuations decreased in both domains (G6PD and 6PGL), as part of this stabilization the loop regions located in the interface of both domains depicted lesser fluctuation (396–411, 495–514) (Figure 8C), these stabilizer effect exerted by NADP^+^ is in agreement with our experimental results, besides it was corroborated that the NADP^+^-G6PG::6PGL complexes are stable along the MD simulation. On another hand, all models studied were stable along the MD simulation and not major modification in domain orientations or linker regions were observed, as reveled by snapshot sampling at 10 ns, 15 ns, and 20 ns (Supplementary Materials Figure S2, 10ns_apo.pdb; 10ns_both.pdb; 10ns_cofactor.pdb, 10ns_structural.pdb, 15ns_apo.pdb; 15ns_both.pdb; 15ns_cofactor.pdb, 15ns_structural.pdb, 20ns_apo.pdb; 20ns_both.pdb; 20ns_cofactor.pdb and 20ns_structural.pdb).

### 3.6. Most Populated Cluster Conformation

**Figure 9:**
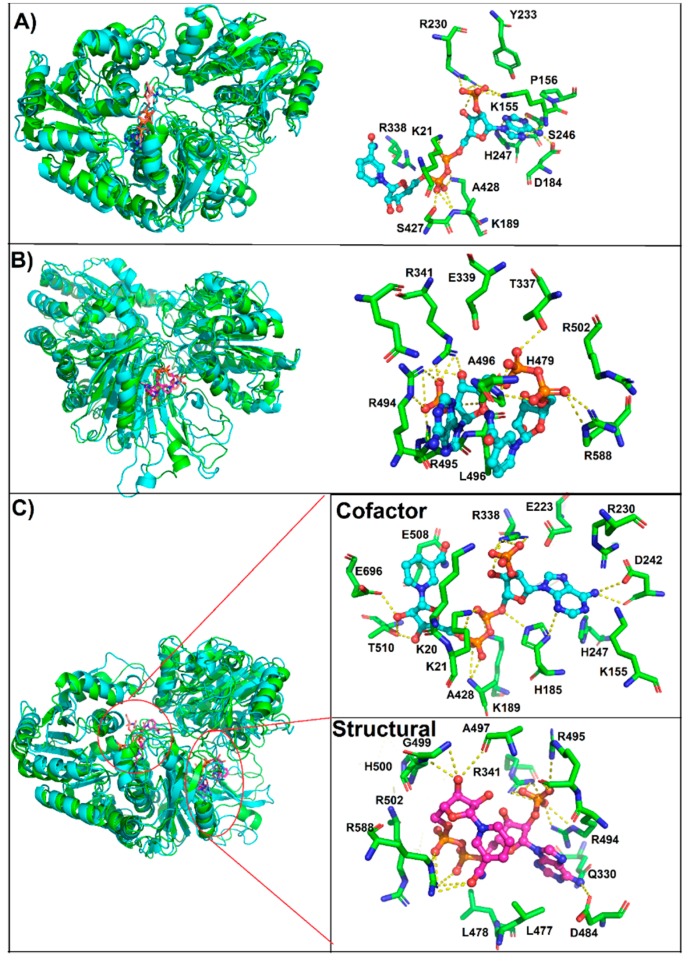
Interactions established in the most populated cluster conformations. (**A**) Cofactor system, (**B**) structural system, and (**C**) holo system. In the left panel, superimposition of the native conformation (green ribbon) with the most populated cluster conformation (cyan ribbon), and in the right panel the NADP^+^ binding mode retrieved from the most populated cluster conformation is depicted; ligand is depicted as ball and sticks while residues as green sticks.

A map of interactions of the most populated cluster conformation obtained by clustering analysis after the MD simulations was produced in order to identify and measure the interactions of NADP^+^ in the G6PD::6PGL structure, cofactor, and Holo systems (Figure 9). As expected, the three systems depicted structural differences regarding the initial conformation; therefore, variations in the interacting residues were observed mainly in the nicotinamide and adenine portions, while phosphate groups were more constantly on the established interactions.

The above geometric parameters from in silico studies and our experimental data indicated that the system was more stable in the presence of both NADP^+^ (holo system), and the interactions established in the most populated cluster conformation of the holo system in the presence of both NADP^+^ are as follows: for cofactor NADP^+^, hydrogen bonds with K21, K155, H185, K189, D242, R338, A428, T510, and E696; and salt bridges with R338 and hydrophobic interactions with K20, K155, P156, E223, R230, H247, and E508, whereas structural NADP^+^ displayed hydrogen bonds with T337, H479, D484, R495, L496, A497, and G499; salt bridges with R341, R494, R502, and R588; and hydrophobic interactions with H500, Q330, L477, and L478. Thus, the interactions obtained by molecular docking were also observed during MD simulation, and more variations were observed in those systems where only one NADP^+^ was studied (structural or cofactor system). Thus, the binding mode observed by docking was also maintained along the MD simulation.

The above geometric parameters from in silico studies and our experimental data indicated that the system was more stable in the presence of both NADP^+^ (holo system). The interactions established in the most populated cluster conformation of the holo system in the presence of both NADP^+^ were as follows: for cofactor NADP^+^, hydrogen bonds with K21, K155, H185, K189, D242, R338, A428, T510, and E696; salt bridges with R338; and hydrophobic interactions with K20, K155, P156, E223, R230, H247, and E508, whereas structural NADP^+^ displayed hydrogen bonds with T337, H479, D484, R495, L496, A497, and G499; salt bridges with R341, R494, R502, and R588; and hydrophobic interactions with H500, Q330, L477, and L478. Thus, the interactions obtained by molecular docking were also observed during MD simulation, and more variations were observed in those systems where only one NADP^+^ was studies (structural or cofactor system). Thus, the binding mode observed by docking was also maintained along the MD simulation.

## 4. Conclusions

In this work, we demonstrated the presence of the structural NADP^+^ binding site on the fused G6PD::6PGL protein from *G. lamblia* and evaluated the effect of this molecule on the stability of the protein. By biochemical and physicochemical assays, we determined that the protein became more resistant to temperature both in its active site (thermal inactivation) and in its thermal stability (global stability of the protein). Furthermore, the enzyme was more resistant to trypsin digestion and a chaotropic agent in presence of the NADP^+^ molecule, and no alterations were observed in the secondary structure in the presence of this molecule. By molecular docking, we determined the possible structural-NADP^+^ binding site in the G6PD::6PGL from *G. lamblia*, which is located between the Rossmann fold of G6PD and 6PGL. Furthermore, it was possible to observe that the binding of the ADP moiety of NADP^+^ is a similar way to that in humans, although the composition was not similar; thus, the structural NADP^+^ binding site of *G. lamblia* was partially conserved between human and other species, as determined above by sequence alignment. By MD simulation, we observed that this complex was stable along the time, suggesting that the presence of both NADP^+^, structural and cofactor, increased the stability of the enzyme; still, these simulations are in agreement with our experimental results. Future X-ray crystal structure determination of fused G6PD::6PGL protein and co-crystallization trials with NADP^+^ will allow us to test the structural findings proposed in this work.

## Supplementary Materials

The following are available online at https://www.mdpi.com/2218-273X/10/1/46/s1, Figure S1: Multiple sequence alignment (MAS) of G6PDs amino acid sequences, Figure S2: Superposition of the snapshots obtained from MD simulations sampling by MD at 5 ns, 10 ns and 20 ns, G6PD::6PGL model (g6pd.6pgl.pdb), G6PD::6PGL model refined (g6pd_clean-rama.pdf), G6PD::6PGL model minimized (Gl-G6PD-6PGL_Minimized.pdb), G6PD::6PGL model docking (docking_both.pdb), Apo-G6PD::6PGL (cluster.apo.pdb), cofactor NADP^+^–G6PD::6PGL (cluster.cofactor.pdb), structural NADP^+^–G6PD::6PGL (cluster.structural.pdb), holo NADP^+^–G6PD::6PGL (cluster.holo_both.pdb), 10ns_apo.pdb; 10ns_both.pdb; 10ns_cofactor.pdb, 10ns_structural.pdb, 15ns_apo.pdb; 15ns_both.pdb; 15ns_cofactor.pdb, 15ns_structural.pdb, 20ns_apo.pdb; 20ns_both.pdb; 20ns_cofactor.pdb and 20ns_structural.pdb).
